# Supplementary material for: Efficacy of a Validated Yoga Protocol on Dyslipidemia in Diabetes Patients: NMB-2017 India Trial
Source: Medicines (Basel). 2019 Oct 11;6(4):100. doi: 10.3390/medicines6040100 (PMC6963794; doi:10.3390/medicines6040100)
Supplement: Supplementary file 1 [file medicines-06-00100-s001.pdf]

Supplementary Materials: Efficacy of a validated Yoga Protocol on Dyslipidemia in Diabetes Patients: NMB-2017 India Trial

Raghuram Nagarathna, Rahul Tyagi, Gurkeerat Kaur , Vetri Vendan, Ishwara N. Acharya, Akshay Anand, Amit Singh and Hongasandra R. Nagendra

Table S1. Effect of yoga intervention on lipid profile in different ranges of HbA1c in urban and rural subjects.

Variables Lipids	(Pre) Mean ± SD	(Post) Mean ± SD	*t* value	*p* value	(Pre) Mean ± SD	(Post) Mean ± SD	*t* value	*p* value
	Rural HbA1c 6.5–8, Cholesterol > 200				Urban HbA1c 6.5–8, Cholesterol > 200			
Total Cholesterol	226.01 ± 23.93	198.47 ± 37.08	7.019	<0.001	230.87 ± 28.83	204.40 ± 44.20	8.393	<0.001
Triglycerides	222.78 ± 99.65	194.67 ± 94.32	2.607	0.011	194.16 ± 84.71	185.82 ± 113.15	1.061	0.290
HDL	50.48 ± 15.34	47.06 ± 11.07	2.343	0.022	51.99 ± 7.65	49.09 ± 11.48	2.165	0.032
LDL	130.85 ± 30.38	111.59 ± 37.66	5.144	<0.001	142.00 ± 30.63	119.96 ± 39.08	7.832	<0.001
VLDL	39.90 ± 17.15	36.46 ± 17.33	1.503	0.137	36.78 ± 14.68	33.51 ± 14.20	2.719	0.007
	Rural HbA1c 8.1–9, Cholesterol > 200				Urban HbA1c 8.1–9, Cholesterol > 200			
Cholesterol	227.37 ± 24.72	195.94 ± 33.96	4.378	<0.001	234.89 ± 29.96	204.48 ± 34.24	5.549	<0.001
Triglycerides	226.50 ± 169.71	219.12 ± 121.31	0.333	0.742	238.43 ± 157.94	201.75 ± 109.34	2.141	0.037
HDL	49.77 ± 12.10	46.43 ± 11.06	1.331	0.192	51.62 ± 21.04	47.76 ± 11.10	1.427	0.159
LDL	135.97 ± 30.28	108.56 ± 32.45	4.353	<0.001	140.76 ± 22.61	117.92 ± 29.04	4.873	<0.001
VLDL	33.29 ± 13.37	34.27 ± 15.13	−0.361	0.721	40.44 ± 16.04	37.22 ± 16.53	1.312	0.196
	Rural HbA1c 9.1–10, Cholesterol > 200				Urban HbA1c 9.1–10, Cholesterol > 200			
Cholesterol	235.24 ± 24.18	199.97 ± 45.31	4.736	<0.001	233.63 ± 24.91	190.96 ± 47.24	5.819	<0.001
Triglycerides	294.13 ± 167.05	218.09 ± 160.49	2.110	0.043	227.04±110.35	202.44 ± 108.02	1.322	0.192
HDL	45.34 ± 10.14	43.00 ± 9.76	1.265	0.215	49.55 ± 12.82	47.55 ± 10.60	1.143	0.258
LDL	135.66 ± 24.27	114.28 ± 27.54	3.974	<0.001	140.24 ± 22.38	106.88 ± 40.64	5.757	<0.001
VLDL	43.34 ± 14.26	34.69 ± 16.73	2.261	0.034	41.06 ± 16.60	35.94 ± 17.07	1.735	0.090
	Rural HbA1c > 10, Cholesterol > 200				Urban HbA1c > 10, Cholesterol > 200			
Cholesterol	243.85 ± 40.56	202.29 ± 52.70	5.502	<0.001	249.90 ± 37.25	210.48 ± 50.86	6.770	<0.001
Triglycerides	282.78 ± 172.00	213.10 ± 125.63	2.882	0.006	281.20 ± 178.78	233.95 ± 148.89	2.453	0.016
HDL	48.84 ± 11.11	48.77 ± 11.25	0.042	0.966	49.56 ± 13.75	46.00 ± 10.95	2.402	0.019
LDL	142.14 ± 33.15	112.00 ± 44.23	4.837	<0.001	146.83 ± 32.03	120.69 ± 40.24	5.211	<0.001
VLDL	42.23 ± 14.42	35.28 ± 14.94	2.821	0.007	44.61 ± 15.58	39.59 ± 18.81	1.843	0.070
